# The effect of nonpharmaceutical interventions on influenza virus transmission

**DOI:** 10.3389/fpubh.2024.1336077

**Published:** 2024-02-08

**Authors:** Danlei Chen, Ting Zhang, Simiao Chen, Xuanwen Ru, Qingyi Shao, Qing Ye, Dongqing Cheng

**Affiliations:** ^1^School of Medical Technology and Informatlon Engineering, Zhejiang Chinese Medical University, Hangzhou, China; ^2^Department of Laboratory Medicine, Children's Hospital, Zhejiang University School of Medicine, National Clinical Research Center for Child Health, National Children's Regional Medical Center, Hangzhou, China

**Keywords:** influenza virus, COVID-19, nonpharmaceutical interventions (NPIs), China, infection

## Abstract

**Background:**

The use of nonpharmaceutical interventions (NPIs) during severe acute respiratory syndrome 2019 (COVID-19) outbreaks may influence the spread of influenza viruses. This study aimed to evaluate the impact of NPIs against SARS-CoV-2 on the epidemiological features of the influenza season in China.

**Methods:**

We conducted a retrospective observational study analyzing influenza monitoring data obtained from the China National Influenza Center between 2011 and 2023. We compared the changes in influenza-positive patients in the pre-COVID-19 epidemic, during the COVID-19 epidemic, and post-COVID-19 epidemic phases to evaluate the effect of NPIs on influenza virus transmission.

**Results:**

NPIs targeting COVID-19 significantly suppressed influenza activity in China from 2019 to 2022. In the seventh week after the implementation of the NPIs, the number of influenza-positive patients decreased by 97.46% in southern regions of China and 90.31% in northern regions of China. However, the lifting of these policies in December 2022 led to an unprecedented surge in influenza-positive cases in autumn and winter from 2022 to 2023. The percentage of positive influenza cases increased by 206.41% (*p* < 0.001), with high positivity rates reported in both the northern and southern regions of China.

**Conclusion:**

Our findings suggest that NPIs against SARS-CoV-2 are effective at controlling influenza epidemics but may compromise individuals’ immunity to the virus.

## 1 Introduction

The influenza virus is a widely spread respiratory pathogen (1). It is prevalent in all countries and poses a significant public health concern globally (2). The World Health Organization estimated that influenza infection affects approximately 1 billion people annually, causing 300,000 to 500,000 fatalities (3). The influenza virus is highly infectious, and all populations are susceptible (4), especially children and the older adult (5). Infection can lead to acute respiratory disease, and severe cases can lead to pneumonia, multiple-organ complications and even death (6, 7). Influenza viruses can be divided into four different types according to their core proteins: A, B, C, and D. The spread of influenza is usually the result of the combined effect of climate factors and population mobility patterns. Low humidity and low temperature are conducive to further spread of influenza (8–10). China is a country with a high incidence of influenza. Every year, there are flu seasons of different severities.

The COVID-19 outbreak in early 2020 presented major obstacles to worldwide public health (11). To mitigate and decelerate the spread of COVID-19, numerous countries have implemented intervention measures. Since January 2020, China has enforced nonpharmaceutical interventions (NPIs), which include quarantining patients, contact tracing, isolating contacts, limiting travel, canceling large gatherings, observing hand hygiene and wearing masks. Like those of COVID-19, influenza viruses are mainly transmitted through the respiratory tract (12, 13). Moreover, NPIs may affect the spread of the influenza virus (14–16). Several studies have shown that just 2 weeks after the United States issued the COVID-19 emergency statement and introduced public health measures, the spread of influenza viruses in the United States dropped sharply. After the implementation of NPIs in Singapore, the percentage of influenza-positive individuals decreased by 64%, the number of daily influenza-positive individuals decreased by 76%, and epidemic activities in Europe, South Korea and other places also decreased (13). However, there has been limited analysis of the epidemiological profile of influenza viruses during the preepidemic, epidemic, and postepidemic phases. Consequently, previous studies have not been able to comprehensively and systematically evaluate the effectiveness of NPIs against SARS-CoV-2 in preventing influenza. We performed a retrospective analysis by aggregating weekly influenza surveillance reports issued by the Chinese Influenza Center over the period from 2011 to 2023. This comprehensive dataset encompasses incidence rates and counts of confirmed influenza cases, in addition to the laboratory-identified subtypes of influenza viruses, spanning three discrete periods: pre-COVID-19 epidemic, during the COVID-19 epidemic, and post-COVID-19 epidemic phases. Our objective was to provide a comprehensive and objective analysis of the impact that the aforementioned epidemic has had on the incidence of influenza in China. This article presents an overview of the epidemiological characteristics of seasonal influenza in the pre-COVID-19 epidemic, during the COVID-19 epidemic, and post-COVID-19 epidemic phases. Specifically, we focused on the alterations that occurred in the epidemiological patterns of influenza during the post-COVID-19 outbreak period to identify more effective strategies for managing and mitigating seasonal influenza transmission.

## 2 Methods

### 2.1 Data and sample sources

Influenza cases reported by the influenza surveillance network of the China National Influenza Center^1^ from 17 October 2011 to 23 April 2023 were used as the research objects. The data included the total number of weekly tests in China, the northern and southern regions of China provided by 554 designated hospitals nationwide, the number of laboratory-confirmed influenza-positive patients, the number of influenza-positive patients, the number of influenza-like illness cases, the proportion of influenza-positive patients, and the number of laboratory subtypes of influenza viruses. Data from Hong Kong, Macau and Taiwan were not included in the study because of significant differences in the data collection methods used in these regions. China’s 31 provinces, autonomous regions and municipalities are divided into southern regions and northern regions (17).

### 2.2 Definition of the influenza cycle and season and the division between north and south China

Temperatures in the northern and southern regions of China begin to drop in mid to late October, and influenza viruses gradually spread through the population, peaking in January or February of the following year (18). In addition, southern regions of China will experience a new peak of outbreaks in the summer (18). To detect a complete influenza peak in an influenza cycle year. Therefore, in this paper, the annual epidemiological cycle was defined as the period from week 42 of the current year to week 41 of the next year. The influenza season and noninfluenza season were defined as follows: the start of the influenza season indicated when the test positivity rate was >10% for two consecutive weeks. If the positivity rate remained <10% for at least three consecutive weeks, the first week with a positivity rate less than 10% was defined as the end week of the influenza season, which is consistent with the findings of the study by Feng L (19).

### 2.3 Statistical analyses

Microsoft Excel software was used to sort and analyze the count data (ratio/composition ratio). The *χ*^2^ test was performed using SPSS 24.0 to compare the differences in detection rates and correlations. GraphPad Prism 9.5 was used to compute the 95% CI median. *p* < 0.05 was considered to indicate statistical significance.

## 3 Results

### 3.1 Epidemiological characteristics of influenza of the pre-COVID-19 epidemic phase in China during 2011–2019, when no NPIs were adopted

**Figure 1:**
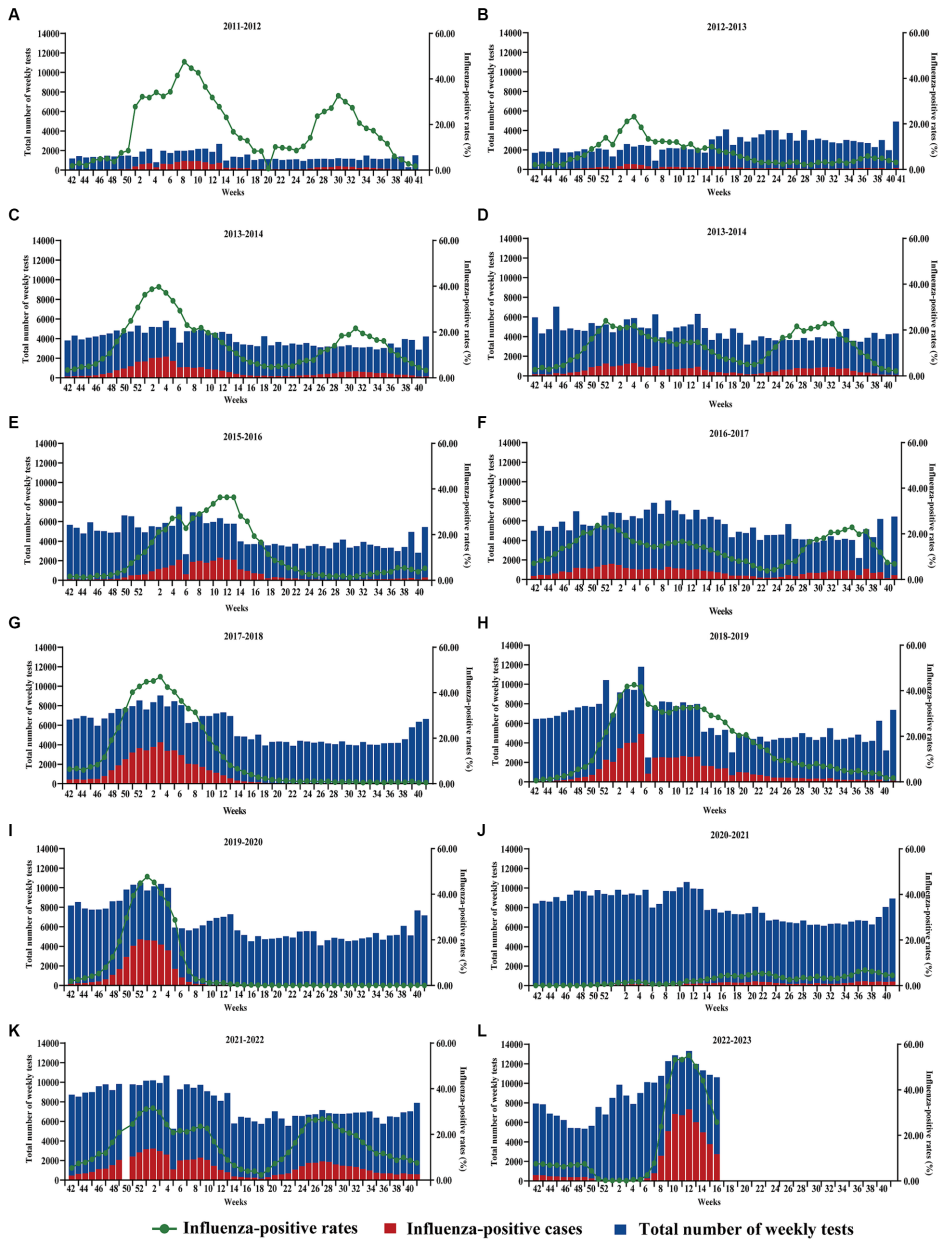
Changes in the total number of tests, the number of influenza-positive patients, and the number of influenza-positive patients in China, 2011–2023. **(A–L)** 2021–2022 Missing data for the 50th week. The horizontal coordinates indicate the beginning of the 42nd week of the previous year and the end of the 41st week of the following year.

**Figure 2:**
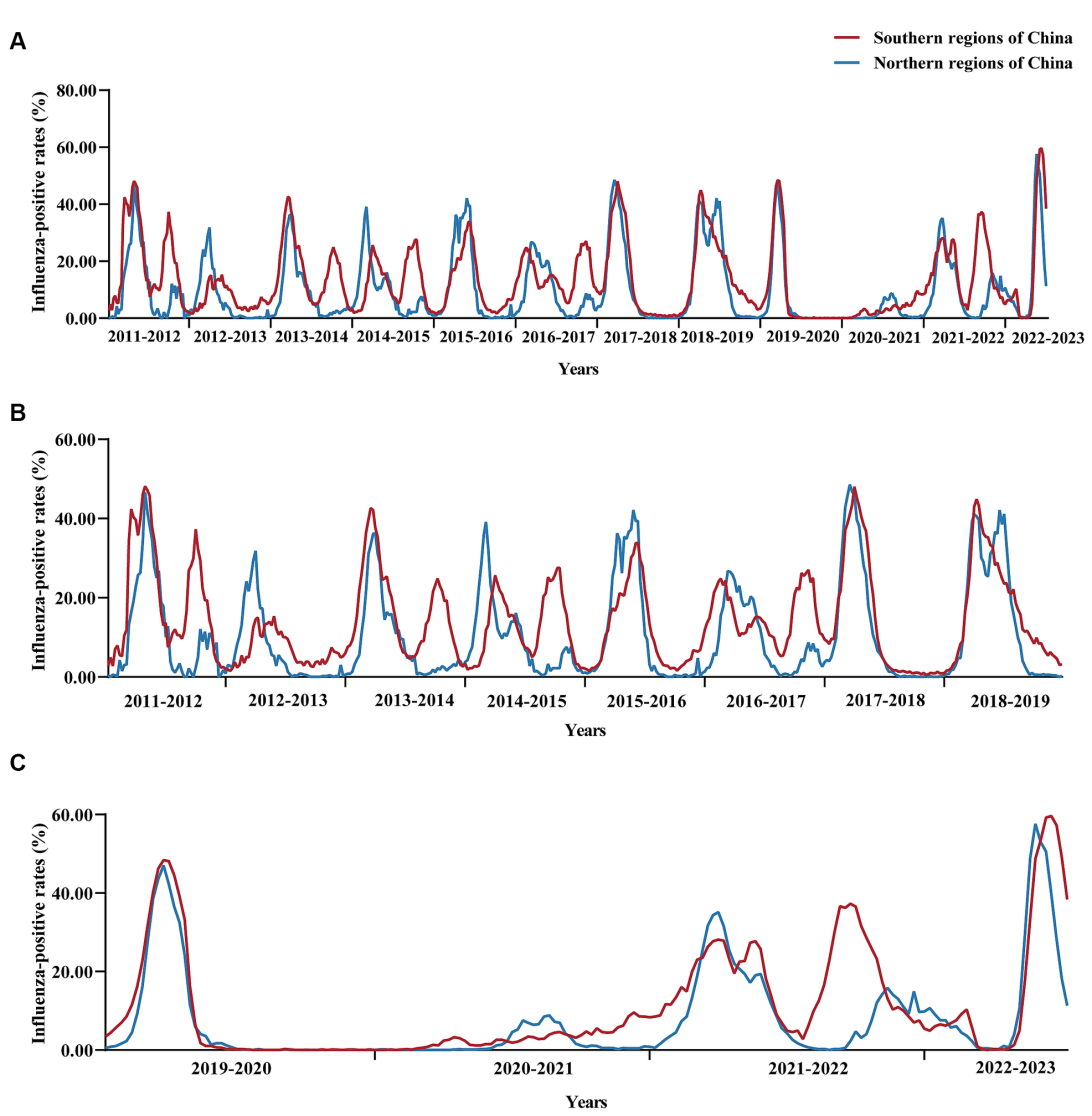
Changes in influenza positivity rates in southern and northern regions of China from 2011 to 2023. **(A)** Changes in the influenza positivity rate from 2011 to 2023, **(B)** Changes in influenza positivity rates from 2011 to 2019 (when NPIs were not implemented during the pre-COVID-19 epidemic), and **(C)** Changes in influenza positivity rates from 2019 to 2023 (during the COVID-19 epidemic and post-COVID-19 epidemic phases). 2021–2022 missing data for the 50th week.

**Figure 3:**
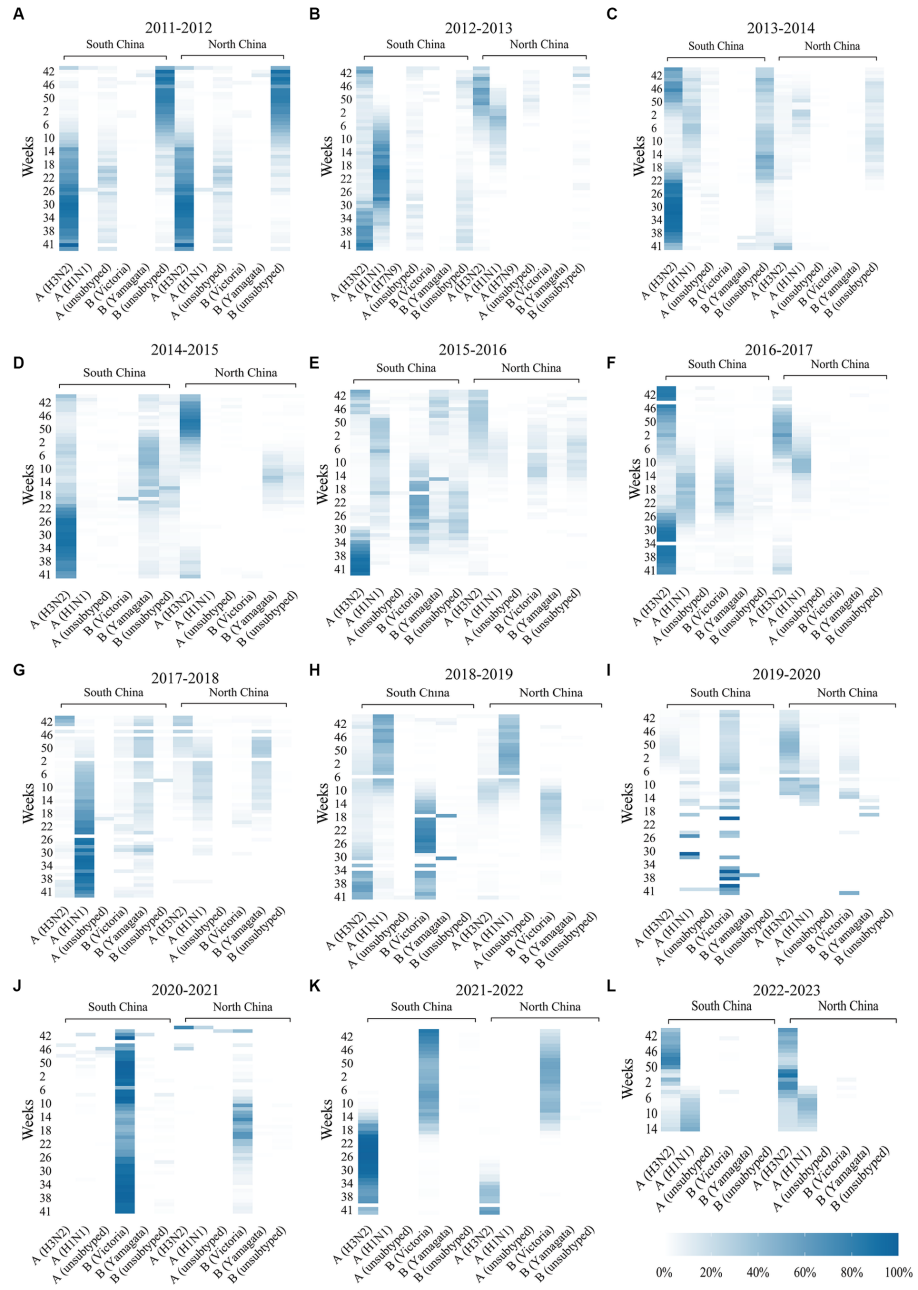
Thermodynamic diagram **(A–L)** of the activity of influenza subtypes in southern regions and northern regions of China during 2022–2023. The dark color in the picture indicates a high proportion and high activity of the influenza subtype. A light color indicates a low proportion and low activity of the influenza subtype. Data were missing for the southern regions and northern regions of the China influenza subtype at week 27 of 2013, week 46 of 2016, week 33 of 2017, week 45 of 2017, week 2 of 2018, week 24 of 2018, week 7 of 2019, week 31 of 2019, week 36 of 2019, week 8 of 2020, week 46 of 2020, week 50 of 2021 and 39 weeks of 2022.

From 2011 to 2019, the incidence of influenza-positive patients in China increased steadily. The percentage of influenza-positive individuals in 2012–2013 was 6.54% (8,868/135,545), and the percentage of influenza-positive individuals in 2018–2019 was 18.41% (58,656/318,543). A significant seasonal trend was observed, with eight winter and spring influenza epidemics and five summer influenza epidemics occurring in China (Figures 1A–H; Table 1). Eight winter and spring influenza epidemics and four summer influenza epidemics occurred in the southern regions of China, and eight winter and spring influenza epidemics occurred in the northern regions of China (Table 1; Figures 2A,B). In the northern regions of China, seasonal influenza epidemics occur in winter and spring, and influenza activity remains low during the remainder of the year. Influenza activity in the southern regions of China was greater than that in the northern regions (Figures 3A–H). Combined with the heatmap, these findings show that many A/H1N1, B/Victoria and B/Yamagata strains are prevalent in the southern regions of China in winter, and A/H3N2 is prevalent in summer. The subtypes of the annual epidemic strains in the northern regions of China are different, and a small epidemic of A/H3N2 occasionally occurs in summer. According to the analysis of the distribution of influenza virus subtypes from 2011 to 2019, influenza A accounted for 60.06% (174,788/264,573) of the viruses, and influenza A was more prevalent and infected more people (Supplementary Figures 1A–H).

**Table 1:** Influenza activity in northern and southern China during 2011–2023 included influenza peak, influenza season start time, end time duration.

			Peak1					Peak 2				
Seasonal year	Region	Peaks	Onset week	Duration, weeks	Peak level, %	End week	Positive rate > 20%, weeks	Onset week	Duration, weeks	Peak level, %	End week	Positive rate > 20%, weeks
2011–2012	South	2	49	20	48.10%	18	14	20	19	37.30%	38	7
	North	1	1	19	46.60%	19	11	32	7	12.10%	38	0
	Total	2	50	21	47.50%	18	14	25	14	32.60%	38	7
2012–2013	South	1	3	16	15.30%	18	0					
	North	1	49	13	31.80%	9	7					
	Total	1	51	18	23.20%	16	2					
2013–2014	South	2	47	21	42.60%	15	15	26	11	24.90%	36	5
	North	1	50	28	36.30%	15	8					
	Total	2	48	20	39.70%	15	12	26	12	21.60%	37	1
2014–2015	South	2	52	16	25.60%	15	5	24	15	27.50%	37	9
	North	1	47	22	39.10%	16	6					
	Total	2	49	20	24.00%	16	5	24	14	22.80%	37	5
2015–2016	South	1	52	22	33.80%	20	12					
	North	1	53	18	42.10%	17	13					
	Total	1	52	21	36.40%	19	14					
2016–2017	South	2	42	31	24.80%	20	7	27	15	26.20%	41	9
	North	1	49	19	26.70%	15	8					
	Total	2	45	26	23.60%	18	6	28	13	22.90%	40	5
2017–2018	South	1	47	19	48.00%	13	15					
	North	1	47	19	48.50%	12	12					
	Total	1	48	19	47.01%	13	13					
2018–2019	South	1	51	27	44.90%	25	21					
	North	1	51	24	41.00%	22	19					
	Total	1	50	31	42.60%	27	21					
2019–2020	South	1	47	13	48.40%	7	9					
	North	1	49	11	47.00%	7	8					
	Total	1	48	12	47.70%	7	8					
2020–2021	South	0										
	North	0										
	Total	0										
2021–2022	South	2	43	22	28.10%	13	12	21	18	37.30%	38	10
	North	2	48	17	35.10%	13	8	32	9	15.80%	40	0
	Total	2	45	21	31.50%	14	13	34	17	27.10%	38	8
2022–2023	South		7		59.60%							
	North		7		57.60%							
	Total		7		55.10%							

### 3.2 Epidemiological characteristics of influenza in China during the COVID-19 epidemic, 2019–2022, when NPIs were adopted

After the COVID-19 outbreak, China implemented NPIs between 2020 and 2022, and the number of influenza-positive cases decreased and then increased during these 3 years. We analyzed the pattern of the influenza season and found that the 2019–2020 influenza season started in the 48th week of 2019 (25 November to 1 December), with an influenza-positive rate of 12.50%. It peaked in the first week of 2020 (30 December 2019 to 5 January 2020), with an influenza-positive rate of 47.7% (Figure 1I; Supplementary Figure 2). Following the COVID-19 outbreak, NPIs were fully implemented in week 2 (January 7, 2020) in most areas of the country. According to the data from the southern regions of China, the percentage of influenza-positive individuals decreased by 50.90% in the third week and 97.46% in the seventh week of NPI implementation. A similar situation was observed in the northern regions of China, where the percentage of influenza-positive individuals decreased significantly, by 90.31%, in week 7 (Figure 2; Table 1). The 2019–2020 influenza season ended in the 7th week of the 2020 season (10–16 February), with an influenza-positive rate of 6.20%. Its duration was only 15 weeks. At the beginning of the 2020–2021 influenza cycle, influenza positivity rates were very low in both the northern and southern regions of China (Figures 1J–K). However, between weeks 10 and 26 of 2021, the number of influenza-positive individuals started to increase slightly. From week 42 of 2021, the influenza positivity rate began to rise continuously, and the autumn-winter influenza season of 2021–2022 began, with a peak positivity rate of 28.10% in the southern regions of China and 35.10% in the northern regions of China. In the 21st week of 2021, the southern regions of China entered the summer influenza season, with a peak positivity rate of 37.3%, while the northern region of China experienced a minor peak, with an influenza-positive rate of 27.1%. The percentage of influenza-positive individuals in the southern region of China remained above 20% for 12 weeks, while in the northern regions of China, it remained above 20% for only 8 weeks (Table 1). Although the influenza peak in the northern regions of China was greater than that in the southern regions, the overall intensity of influenza was lower in the northern regions. Overall, both the northern and southern regions of China experienced two influenza peaks (Figure 2C).

During the 2019–2020 epidemic cycle, the A/H3N2 and B/Victoria strains were predominant in the southern regions of China, and the percentages of these two subtypes were 52.31% (10,326/19,740) and 41.18% (8,128/19,740), respectively (*p* < 0.001). In the northern regions of China, the predominant influenza subtypes were A/H3N2, followed by A/H1N1 and B/Victoria, accounting for 78.64% (13,353/16,980), 11.87% (2,015/16,980) and 9.17% (1,557/16,980), respectively (*p* < 0.001). In addition, beginning in the 35th week, the national influenza virus detection rate was mainly B/Victoria, and the detection rates of A and B/Yamagata began to decline. A total of 56 patients with B/Yamagata were detected in the 2019–2020 cycle, a decrease of 75.44% compared with that in the previous cycle (*p* < 0.001). Influenza A accounted for 63.04%, *p* < 0.001 (Figure 3I). In the 2020–2021 cycle, B/Victoria strains predominated in both the northern and southern regions of China, accounting for 97.20% (10,063/10,353) of the detected cases. B/Yamagata was detected in 32 patients, a decrease of 42.86% compared to that in the previous cycle and 85.96% compared to that in the pre-COVID-19 epidemic (*p* < 0.001; Figure 3J). During the 2021–2022 cycle, the main influenza virus subtypes circulating in China were A/H3N2 and B/Victoria. Growth of the A/H3N2 and B/Victoria subtypes has been detected in both southern and northern regions of China. According to the heatmap (Figure 3K), the B/Victoria strain was endemic in autumn and winter, while the A/H3N2 strain was endemic in summer. In addition, no cases of B/Yamagata were detected during the 2021–2022 cycle (Supplementary Figure 1K).

### 3.3 Epidemiological characteristics of influenza in the post-COVID-19 epidemic phases in China, 2022–2023, when NPIs were eliminated

**Figure 4:**
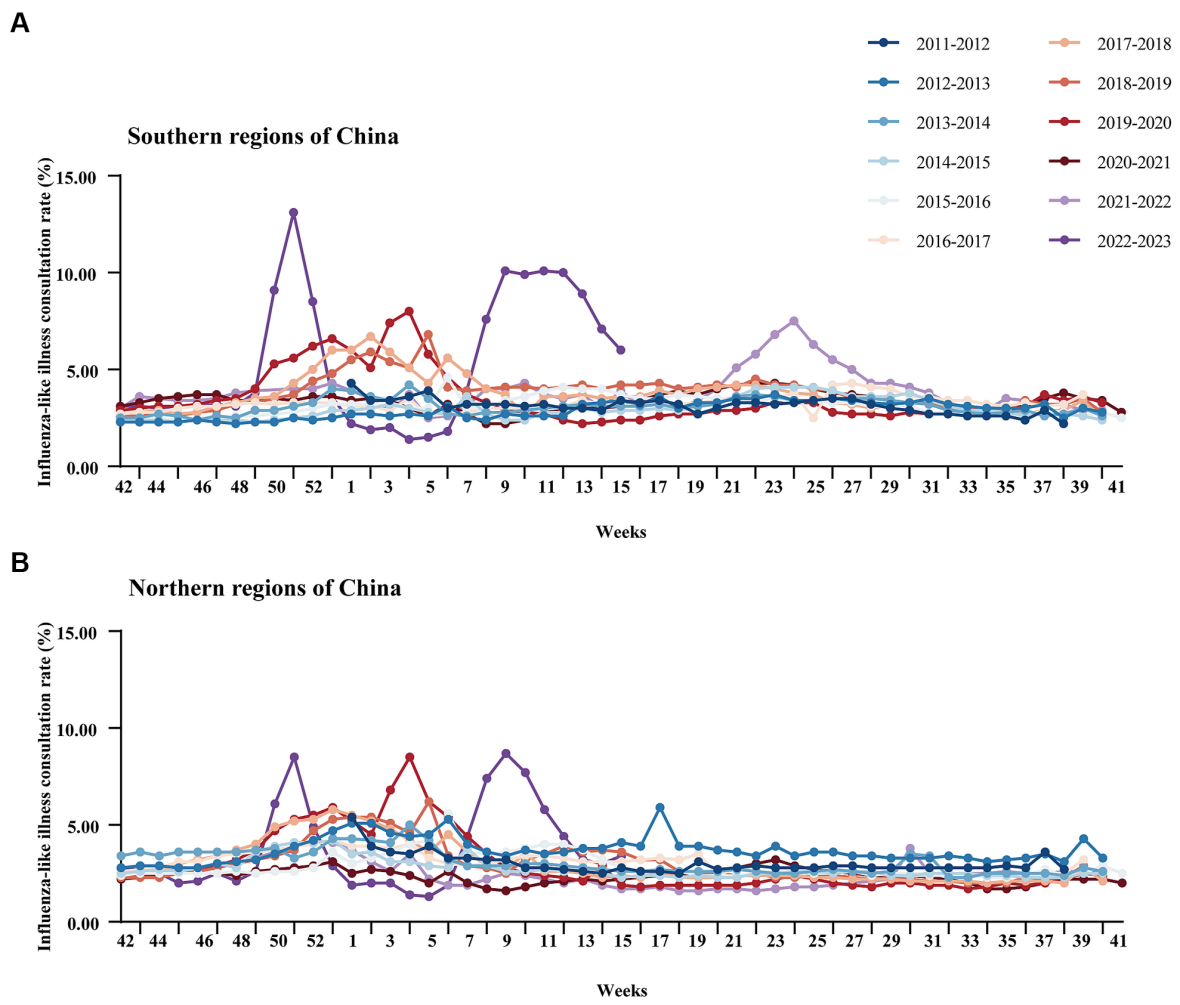
Changes in influenza-like cases in sentinel hospitals in southern regions and northern regions of China during 2011–2023. **(A)** Changes in influenza-like cases in sentinel hospitals in southern regions of China during 2011–2023. **(B)** Changes in influenza-like cases in sentinel hospitals in northern regions of China during 2011–2023. The data were missing from week 42 to week 3 in 2011, week 41 to 42 in 2017, and week 50 in 2021.

**Figure 5:**
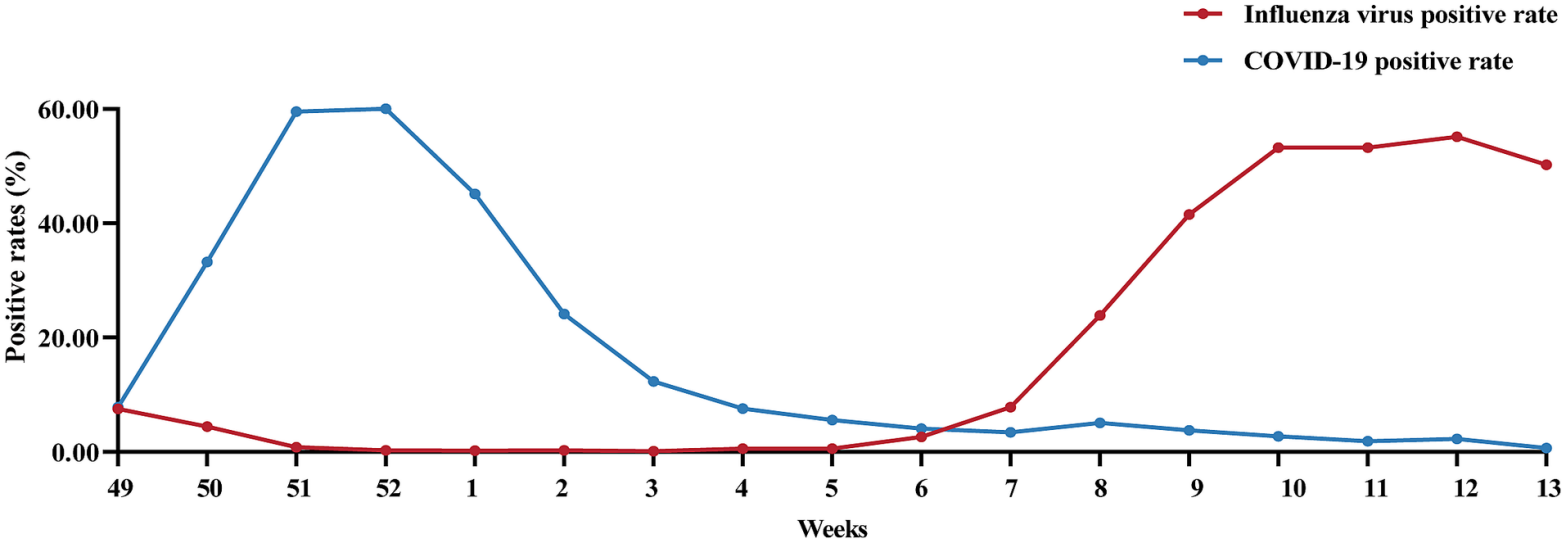
Changes in the percentage of patients positive for influenza virus and SARS-CoV-2 after the cancelation of NPIs in China after December 7, 2022.

In week 49 of 2022 (December 7), following the cancelation of NPIs, the number of confirmed cases of COVID-19 increased sharply. According to the Chinese Centre for Disease Control and Prevention, SARS-CoV-2 positivity peaks at 29.2% in week 52 of 2023 (26 December 2022 to 1 January 2023). Thereafter, the SARS-CoV-2 positivity rate began to fluctuate. In week 5 (30 January to 5 February), the percentage of patients positive for SARS-CoV-2 decreased to a low value of 1.2%. During this period, SARS-CoV-2 spread widely in China, while influenza activity was suppressed. The percentage of influenza-positive patients in China declined rapidly beginning at week 49 (December 5–11), from 7.5 to 0.1% (95% CI median, 0.20% to 4.40). However, the rate of influenza-like cases in outpatients at sentinel hospitals in southern and northern China has increased rapidly and is well above the level for the same period in the previous cycle (Figure 4). This may be related to SARS-CoV-2 infection, when people took protective measures, such as reducing outdoor activities and wearing masks, which affected the spread of the influenza virus. As shown in Figure 5, after the SARS-CoV-2 positivity rate decreased to a low level, the incidence of influenza virus infections began to increase rapidly. According to the latest data from the Chinese Centre for Disease Control and Prevention, China entered the influenza season on week 7 (13–20 February), during which the percentage of positive influenza cases increased by 206.41% in just 1 week (*p* < 0.001). The percentage of influenza-positive patients in sentinel hospitals worldwide has continued to increase, reaching 53.2% at week 10 (6–12 March). In week 12 (20–26 March), the influenza case positivity rate peaked at 55.5% and then began to decline (Figure 1L). The correlation coefficient between the incidence of influenza and the incidence of COVID-19 was −0.531 (*p* = 0.028). This indicates that there is a significant negative correlation between these two variables (*p* < 0.05). By week 15, 2023, a total of 48,527 influenza-positive patients had been detected in China, with influenza A accounting for 99.84% of the patients. According to the heatmap (Figure 3), before the NPIs were lifted, the subtype of the main influenza virus was A/H3N2, accounting for 99.15% of the cases. After the NPIs were lifted, both A/H1N1 and A/H3N2 circulated simultaneously, and the intensity of influenza A H1N1 was greater than that of influenza A H3N2 (Figure 3L; Supplementary Figure 1). In addition, the B/Yamagata subtype was not detected in the 2022–2023 cycle.

## 4 Discussion

A/H1N1 and influenza B were the predominant endemic subtypes during the fall and winter seasons in China. A/H3N2 is endemic in the fall and winter seasons and summer seasons in the southern regions of China, usually during a half-yearly cycle. In northern regions of China, A/H3N2 is predominantly endemic in the fall and winter seasons, generally on a one-year cycle. However, there is no obvious regularity in the subtype prevalence of influenza B, and studies have shown that influenza B usually occurs annually in alternation, with a single influenza B predominating without any obvious regularity, which is in agreement with our conclusions (20). By comparing the annual infection rates of influenza A and influenza B in China, we found that the severity of influenza A is greater than that of influenza B (21). The reason may be that influenza A spreads not only among humans but also infects animals. The influenza A virus can undergo horizontal gene transfer, which can cause repeated infection of the host (22, 23). Influenza B can infect humans and seals, and it has been shown to infect pigs (24, 25). However, meaningful transmission of influenza B viruses from these nonhuman species does not occur very often (25). It can be assumed that these viruses circulate mainly in humans and lack genetic recombination between human influenza viruses and animal influenza viruses; therefore, they do not easily cause a pandemic in humans (26, 27). We also analyzed influenza activity in the northern and southern regions of China and found that influenza activity in the southern region was more active throughout the year. Seasonal influenza in the northern regions of China occurs mainly in the fall and winter seasons and is more stable at other times, showing a trend toward high incidence in the southern regions of China and low incidence in northern China (28).

The COVID-19 pandemic that broke out in early 2020 posed an enormous challenge to global public health. As of March 31, 2020, more than 750,890 cases had been confirmed, resulting in 36,405 deaths (29). In our previous study, we found that the implementation of NPIs in China significantly reduced the transmission of common respiratory viruses among children (15, 30). In this study, we found that the 2019–2020 influenza season ended 8–12 weeks earlier than did the previous cycles, and the number of infections decreased (31, 32). From 2019 to 2021, influenza activity decreased, and influenza transmission was completely suppressed without significant pandemic peaks. From 2022 onwards, influenza activity increased, but the intensity of activity was still lower than that pre-COVID-19 epidemic. These findings suggested that China’s NPI has been effective at limiting the spread of influenza. Studies in other countries have reached the same conclusion. A US study showed that in the first 10 weeks after the implementation of NPIs, the incidence of influenza A (H1N1) and influenza B (H1N1) epidemics in the U.S. at the beginning of 2020 decreased by more than 60% (33–35). In the first wave of the outbreak in 2019, NPIs was used in New Zealand to control the spread of SARS-CoV-2. Using multiple monitoring systems, it was observed that the number of influenza and other respiratory virus infections in 2020 decreased unprecedentedly (36, 37). The COVID-19 pandemic in Europe led to the early end of the influenza season and reduced the spread of influenza viruses (38).

Notably, widely implemented NPIs may lead to a decrease in the epidemic diversity of influenza viruses. Research shows that the detection rate of B/Yamagata has decreased significantly since April 2020 and is approximately 99% lower than that in previous cycles. Since 2019, our B/Victoria detection rate has been increasing, with a high B/Victoria detection rate of 97.20% in 2020–2021 (*p* < 0.001; Supplementary Figure 1). A study in the United States also revealed an abnormal early surge of influenza B cases in 2019–2020, accompanied by an evolutionary shift within the dominant B/Victoria (39). Combined with the monoepidemic pattern of influenza B, we speculate that B/Yamagata may have a period of low prevalence in the pre-COVID-19 epidemic. Widespread implementation of NPIs has reduced the number of international tourists, leading to a decrease in the number of people infected with B/Yamagata (40, 41).

In the past 3 years, the widespread implementation of NPIs has greatly reduced the intensity of influenza virus infections. This may be beneficial in the short term, but research shows that immunization debt may have a greater negative impact (42). From 2019 to 2022, the activity of the influenza virus will be unprecedentedly suppressed. There is insufficient immune stimulation for people infected with influenza virus. When the susceptibility of the population increases, group immunity decreases, increasing the proportion of the population vulnerable to virus infection (43). As of April 25, 2023, the national influenza detection rate was 20.92% (48,527/231918), and the highest positive rate was 55.10%. The peak stage influenza positivity rate was higher than that prior to contracting COVID-19, and the highest level of influenza positivity has occurred since 2011. The 2023 influenza season is more severe than it was in previous years, with high influenza positivity rates that will lead to mass population infections in the near term. Schools were closed in many places, and hospitals saw a multifold increase in flu patients.

In conclusion, this study revealed that the low level of influenza activity in China from 2019 to 2021 was unprecedented, possibly due to the implementation of NPIs. This discovery has been confirmed in the United States and other studies. The high level of influenza activity in China in the fall and winter of 2022–2023 is likely a result of immune debt. We summarized our experience with the COVID-19 outbreak. We found that we should spontaneously adopt nonpharmacological interventions, such as washing hands frequently, wearing masks and reducing people’s movement. Moreover, we can increase the influenza vaccination rate to minimize the negative impact of the outbreak. The weakness of this study is that the association between age and influenza was not analyzed. Future studies could focus on analyzing the disease burden of influenza in different age groups.

## Data availability statement

Publicly available datasets were analyzed in this study. This data can be found at: https://ivdc.chinacdc.cn/cnic/.

## Author contributions

DLC: Writing – original draft, Writing – review & editing. TZ: Writing – original draft, Writing – review & editing. SC: Writing – original draft, Writing – review & editing. XR: Writing – original draft, Writing – review & editing. QS: Writing – original draft, Writing – review & editing. QY: Writing – review & editing. DQC: Writing – review & editing.

## Footnotes

^1^ https://ivdc.chinacdc.cn/cnic/zyzx/lgzb/
