# Supplementary figures and images for: The effect of nonpharmaceutical interventions on influenza virus transmission
Source: Front Public Health. 2024 Feb 8;12:1336077. doi: 10.3389/fpubh.2024.1336077 (PMC10881707; doi:10.3389/fpubh.2024.1336077)

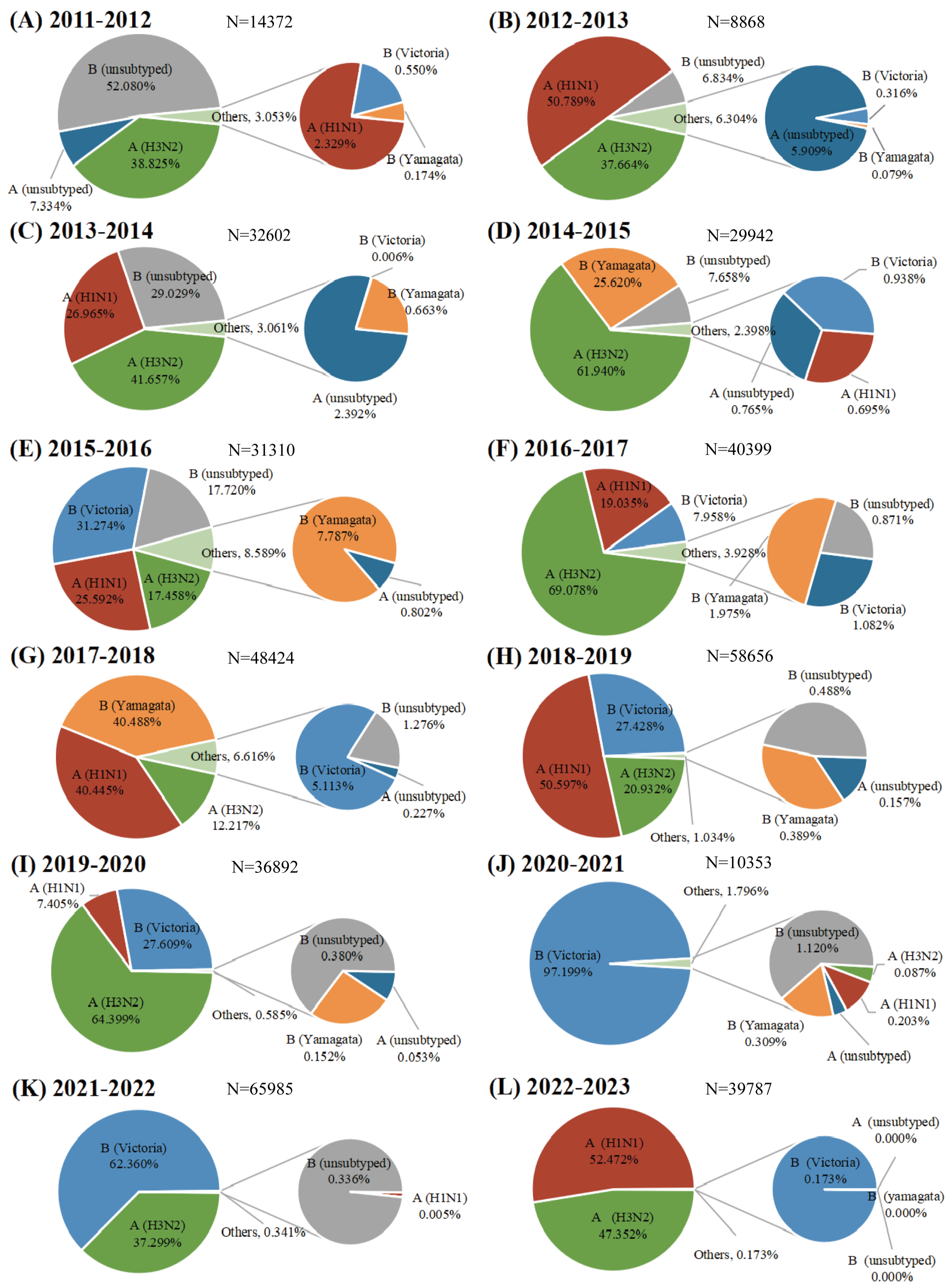

Supplement: Supplementary file 1 [file Image_1.TIF]

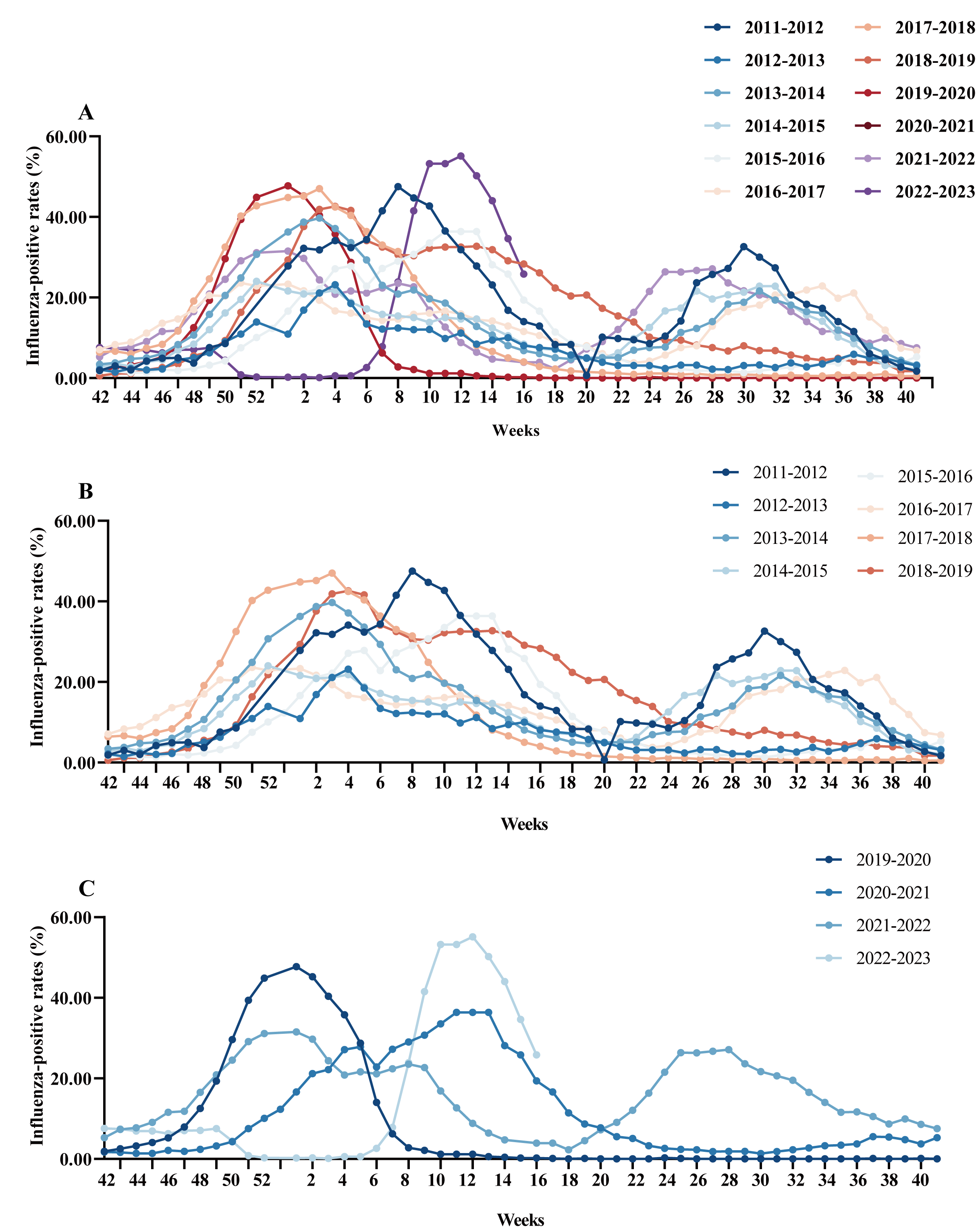

Supplement: Supplementary file 2 [file Image_2.TIF]
